# Giant mediastinal neuroendocrine tumor successfully resected after transarterial chemoembolization of drug-eluting embolic microspheres: A case report

**DOI:** 10.1097/MD.0000000000028247

**Published:** 2021-12-23

**Authors:** Qi Yu, Zhen Li, Xinwei Han

**Affiliations:** Department of Interventional Radiology, the First Affiliated Hospital of Zhengzhou University, No.1, East Jian She Road, Zhengzhou, Henan Province, China.

**Keywords:** drug-eluting embolic, neuroendocrine tumor, transcatheter arterial chemoembolization

## Abstract

**Rationale:**

Neuroendocrine tumors (NETs) in the mediastinum are extremely rare. No uniform solution currently exists for the treatment of mediastinal NETs.

**Patient concerns:**

We report the case of a 32-year-old man with symptoms of chest tightness, chest pain, cough, and panic.

**Diagnoses:**

Computed tomography showed that the mediastinum and right lung occupied a space with uneven enhancement. A needle biopsy revealed mediastinal NETs. An atypical carcinoid was diagnosed using immunohistochemistry.

**Interventions:**

The patient underwent 2 similar transarterial chemoembolizations of drug-eluting embolic microsphere procedures after 5 cycles of etoposide and cisplatin chemotherapy. The patient underwent successful surgical resection 2 months after the operation.

**Outcomes:**

The patient's quality of life was significantly improved, without chest tightness, chest pain, or other symptoms. At the 1-year follow-up, the patient had no tumor recurrence.

**Lessons:**

For large mediastinal NETs with poor chemotherapy effects, surgical resection is safe and feasible after down-staging treatment via arterial chemoembolization of drug-eluting embolic microspheres.

## 1 Introduction

Neuroendocrine tumors (NETs) are thought to arise from cells throughout the diffuse endocrine system. NETs comprise a broad family of tumors, the most common of which are the gastrointestinal tract, lungs, thymus, and pancreas. Primary mediastinum is extremely rare.^[1]^ No uniform solution currently exists for the treatment of NETs; however, surgical resection is still the standard treatment.^[2]^ Here, a summary and analysis of clinical data is presented for a patient with a giant mediastinal NET that was successfully resected after the descending stage by drug-eluting embolic transcatheter arterial chemoembolization (DEE-TACE) had been performed. No tumor recurrence was observed at the 1-year follow-up after surgical resection.

## 2 Case report

**Figure 1:**
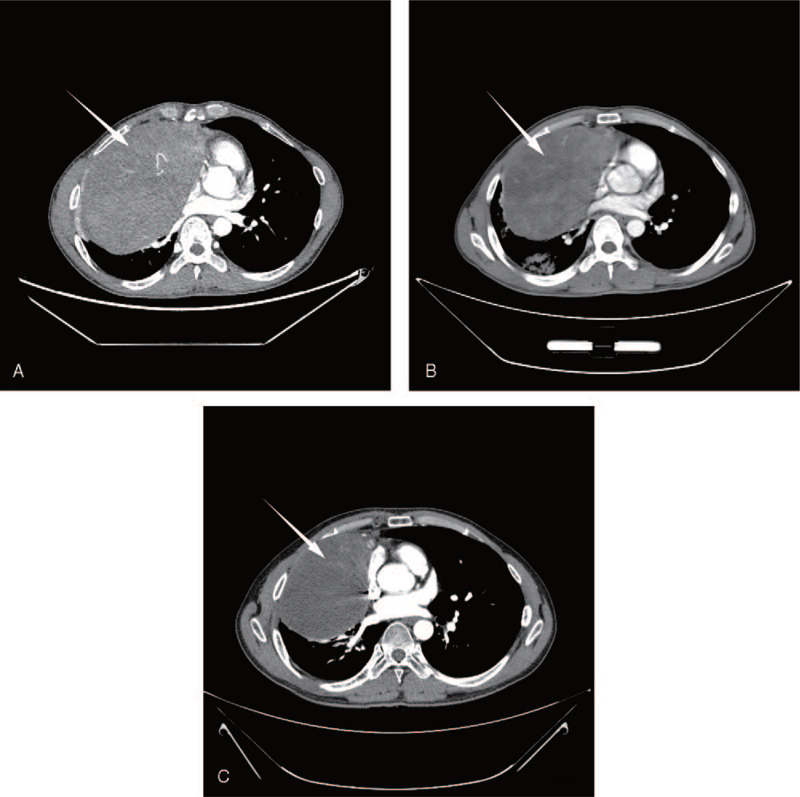
(A) A 11 cm × 17 cm mass located in the right lung and mediastinum. (B) 3 months after chemotherapy, CT scan showed that the mass had shrunk (10 cm × 14 cm). (C) CT image obtained after the third DEE-TACE revealed a considerable tumor reduction to 8.5 cm × 13 cm in size and complete necrosis of the tumor (arrow).

**Figure 2:**
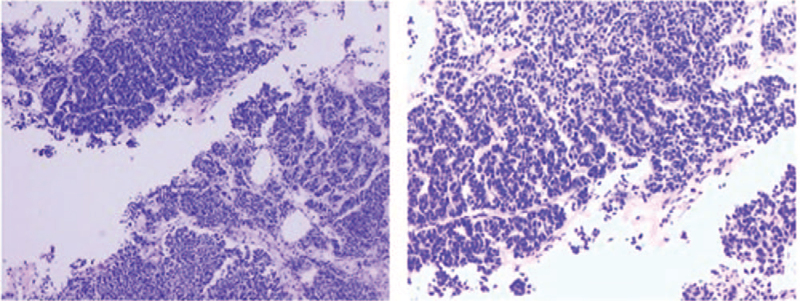
Immunohistochemistry revealed AE1/AE3(CK)(+), CD56(56C04)∗(+), SYN(+), CgA(+), CD117(+), Ki-67(5%+) (H&E, 100×).

A 32-year-old male patient was admitted with symptoms such as chest tightness, chest pain, cough, and panic. Computed tomography (CT) showed that the mediastinum and right lung occupied a space measuring approximately 11 cm × 17 cm, with uneven enhancement (Fig. 1A). A needle biopsy revealed mediastinal NETs. A diagnosis of atypical carcinoid was established using immunohistochemistry (Fig. 2). Immunohistochemistry revealed AE1/AE3(CK)(+), CK5/6(-), CD56(56C04)∗(+), SYN(+), CgA(+), TTF-1(-), NapsinA(-), P63(-), CD117(+), LCA(CD45)(-), Ki-67(5%+). Then, etoposide and cisplatin chemotherapy were administered for 5 cycles. After 3 months, the CT scan revealed that the tumor was slightly smaller than before, measuring approximately 10 cm × 14 cm, but uneven enhancement was still observed (Fig. 1B). Multidisciplinary consultation showed that the tumor was huge and close to the pericardium, so it was difficult to remove. Considering the abundant blood supply to the tumor, surgical resection after TACE was deemed feasible.

**Figure 3:**
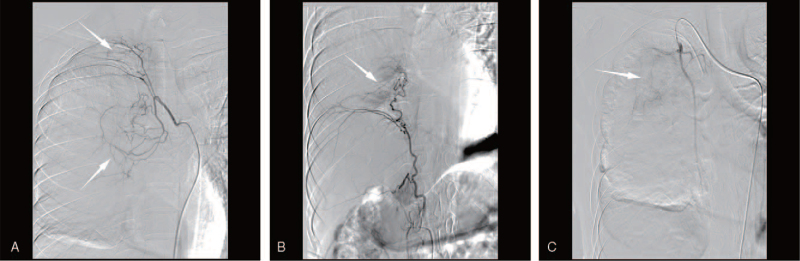
Angiographic image during the first DEE-TACE showed the bronchial artery (A), phrenic artery (B) and internal thoracic artery (C) as a tumor-feeding vessel (arrow).

Using a transfemoral approach, a 5-F Cobra (Cook Medical Products, Bloomington, IN) catheter was used to examine the bronchial artery, internal thoracic artery, and phrenic artery for angiography (Fig. 3). The responsible vessels were then intubated with a 2.7-F PROGREAT microcatheter (Terumo Interventional Systems, Tokyo, Japan). Using a microcatheter, 40 mg of docetaxel and a vial of 300 to 500 μm diameter CalliSpheres microspheres (Suzhou Hengrui Callisyn Biomedical Co., Ltd., Suzhou, Jiangsu Province, China) loaded with 100 mg of oxaliplatin were injected to occlude each tumor artery until angiographic tumor staining disappeared. Then, a 5-F vertebral catheter (Cook Medical Products, Bloomington, IN) was used to intubate the right internal mammary artery, where the tumor staining area could be visualized by angiography. Embolization was subsequently performed using the method described above. It must be noted that the embolic agent should be injected slowly to avoid nontargeted embolization. The patient was administered symptomatic treatment after surgery to reduce edema and prevent infection.

One month after embolization, CT showed a considerable reduction in tumor size and blood supply. After a further round of DEE-TACE consolidation treatment had been completed, the CT scan showed a considerable reduction in tumor size (8.5 cm × 13 cm) and complete necrosis of the tumor (Fig. 1C). After the surgical consultation, the tumor load was significantly reduced, so it was decided to move forward with tumor resection and right upper lobectomy. Postoperative pathology revealed a mediastinal NET along with chronic inflammation of the upper lobe of the right lung and interstitial fibrosis. One month after resection, the CT scan showed no residual tumor. The patient's quality of life was significantly improved, without chest tightness, chest pain, or other symptoms. At the 1-year follow-up, the patient had no tumor recurrence.

## 3 Discussion

NET mainly occurs in the stomach, intestine, and pancreas, but primary mediastinum is rare. Because the symptoms and signs of NET are not typical, their clinical manifestations are complex. Most patients have local spread or distant metastasis when diagnosed, and they lose the chance for radical surgery.^[3]^ The treatment strategy for mediastinal NET is still inconclusive, but surgical resection remains the standard treatment. For patients with highly and moderately differentiated (G1/G2) tumors that cannot be surgically removed and with locally advanced and distant metastases, a combination of systemic and local therapies should be adopted. In this case report, the tumor was considered to be at the G2 stage. An etoposide and cisplatin regimen was used for chemotherapy, but the effect was not good. Considering that the tumor was huge, surrounded by blood vessels, and difficult to surgically remove, embolization was decided to quickly reduce the tumor burden.

TACE has been previously used for the treatment of liver cancer, and this technology has been widely used in extrahepatic tumors.^[4]^ Drug-eluting embolic microspheres can be loaded with chemotherapy drugs; therefore, they can embolize the tumor blood vessel bed and release drugs. According to the National Comprehensive Cancer Network guidelines for NETs, etoposide/cisplatin or etoposide/carboplatin are used as first-line drugs.^[5]^ There is no clear plan for second-line treatment, but fluorouracil or capecitabine combined with oxaliplatin or irinotecan can be considered.^[6]^ Furthermore, oxaliplatin is the only platinum drug that can be loaded with CalliSpheres microspheres. Microspheres that ranged from to 300 to 500 μm in diameter were chosen to avoid possible fistula or dangerous anastomosis.

In conclusion, NETs originating from the mediastinum have rarely been reported. DEE-TACE downstaging treatment for mediastinal NETs may be safe and effective.

## Author contributions

**Conceptualization:** Zhen Li, Xinwei Han.

**Formal analysis:** Zhen Li, Xinwei Han.

**Supervision:** Zhen Li, Xinwei Han.

**Writing – original draft:** Qi Yu, Zhen Li.

**Writing – review & editing:** Zhen Li, Xinwei Han.
